# Non-traumatic Intramural Duodenal Hematoma Secondary to Alcoholic Pancreatitis

**DOI:** 10.7759/cureus.94500

**Published:** 2025-10-13

**Authors:** Rachel M Hernandez, Kevin P Eells, Kyle J Iverson

**Affiliations:** 1 General Surgery, Keesler Medical Center, Biloxi, USA; 2 General Surgery, Rocky Vista University College of Osteopathic Medicine, Parker, USA

**Keywords:** alcoholic pancreatitis, duodenal hematoma, duodenal mass, endoscopic ultrasound (eus), spontaneous intramural hematoma

## Abstract

Duodenal hematomas are a rare phenomenon, with the most common etiology being traumatic in origin. We describe the case of a spontaneous intramural duodenal hematoma in a 56-year-old male as a complication of alcohol-induced pancreatitis. Initial endoscopy showed evidence of a bulging deformity in the second and third parts of the duodenum. Further imaging using endoscopic ultrasound (EUS) demonstrated a round intramural cystic lesion in the third portion of the duodenum that was hypoechoic, septated, and measuring approximately 6.0 x 7.6 cm in diameter and yielded a bloody aspirate. A computed tomography angiography (CTA) of the abdomen and pelvis after aspiration depicted an intramural/periduodenal fluid collection with rim enhancement measuring 12.4 x 10.6 x 6.5cm extending from the proximal duodenum to the distal horizontal segment with possible breach of the inferior serosal wall. Upon attempt of oral intake, the patient’s abdominal pain returned, and subsequent imaging revealed the intramural collection was larger compared to previous imaging. The patient was transferred to a tertiary facility in stable condition. Repeat imaging at the outside facility indicated a newly dilated common bile duct in addition to the intramural hematoma. An AXIOS stent placement (Boston Scientific, Marlborough, Massachusetts, US) was ultimately placed, and the patient was discharged home after three weeks on a clear liquid diet with total parental nutrition (TPN) supplementation. The patient subsequently developed duodenal stenosis secondary to ulceration, resulting in a gastric outlet obstruction, and ultimately underwent a robotic gastrojejunostomy for duodenal bypass eight months after initial presentation.

## Introduction

Duodenal hematomas are an uncommon occurrence, with a low rate of incidence in both pediatric and adult populations. Duodenal hematomas are classified based upon their etiology, traumatic or non-traumatic, where blunt trauma accounts for approximately 70% [1,2]. Even more rare is the development of spontaneous hematomas; however, their occurrence is being increasingly reported within the last decade. With the increase in anticoagulation therapy, there has been a steady increase in the incidence of duodenal hematomas; however, there are other etiologies as well. Other predisposing factors include, but are not limited to, coagulopathic states, lymphoma, chemotherapy, vasculitis, endoscopic procedures, pancreatitis, or pancreatic malignancies [2]. We present a case of a spontaneous intramural duodenal hematoma in the setting of acute alcoholic pancreatitis.

## Case presentation

A 56-year-old male with a past medical history significant for alcohol misuse disorder presented to an outside hospital for a 3-day history of nausea, vomiting, and inability to tolerate oral intake after a recent binge drinking episode. Outside workup demonstrated a duodenal cyst measuring approximately 10 x 4.5 cm, causing an obstruction with evidence of upstream dilation. The patient was then transferred to our community hospital for gastroenterology capabilities. Upon arrival to our emergency department, the patient was afebrile, hypertensive to 191/108mmHg, heart rate 86 bpm, tachypnea at 22 bpm, and saturating at 99% on room air. He reported abdominal discomfort with 10/10 abdominal pain; however, his abdomen was soft, with mild distention and moderate tenderness to palpation in the epigastric and right upper quadrant; however, there was no evidence of peritonitis. Review of laboratory studies was normal, including a coagulation panel, with the only notable findings of mild hypokalemia (2.9 mmol/l) and an elevated lipase (16890 u/l). Gastroenterology was consulted and recommended EGD with endoscopic ultrasound for further evaluation.

**Figure 1:**
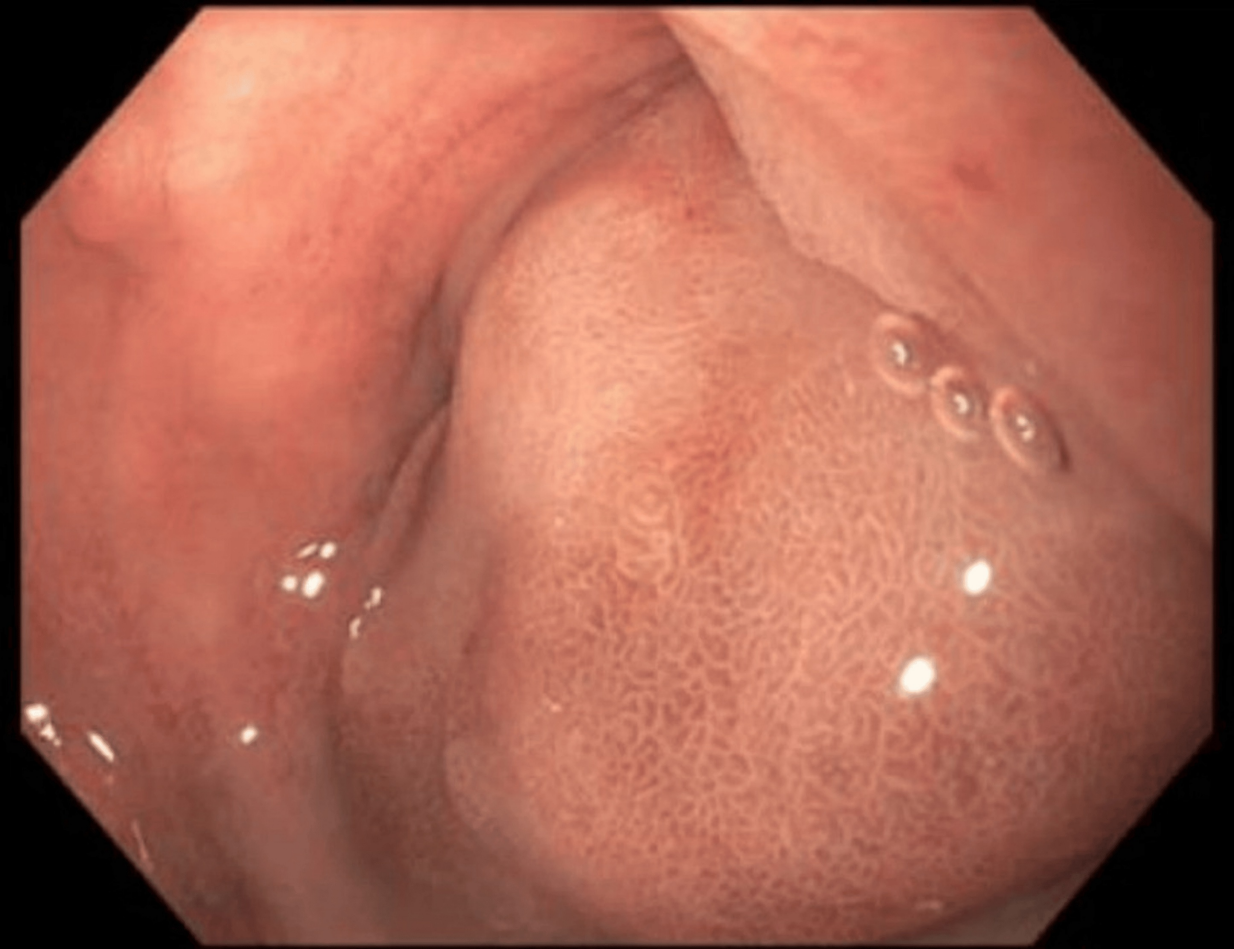
Endoscopy demonstrating an obstructing mass in the second and third portions of the duodenum

On endoscopy, there was LA Grade A esophagitis without bleeding at the gastroesophageal junction with retained fluid in the gastric body. There was evidence of a bulging deformity with edematous mucosa in the second and third portions of the duodenum, with normal mucosa in the fourth portion of the duodenum (Figure 1).

**Figure 2:**
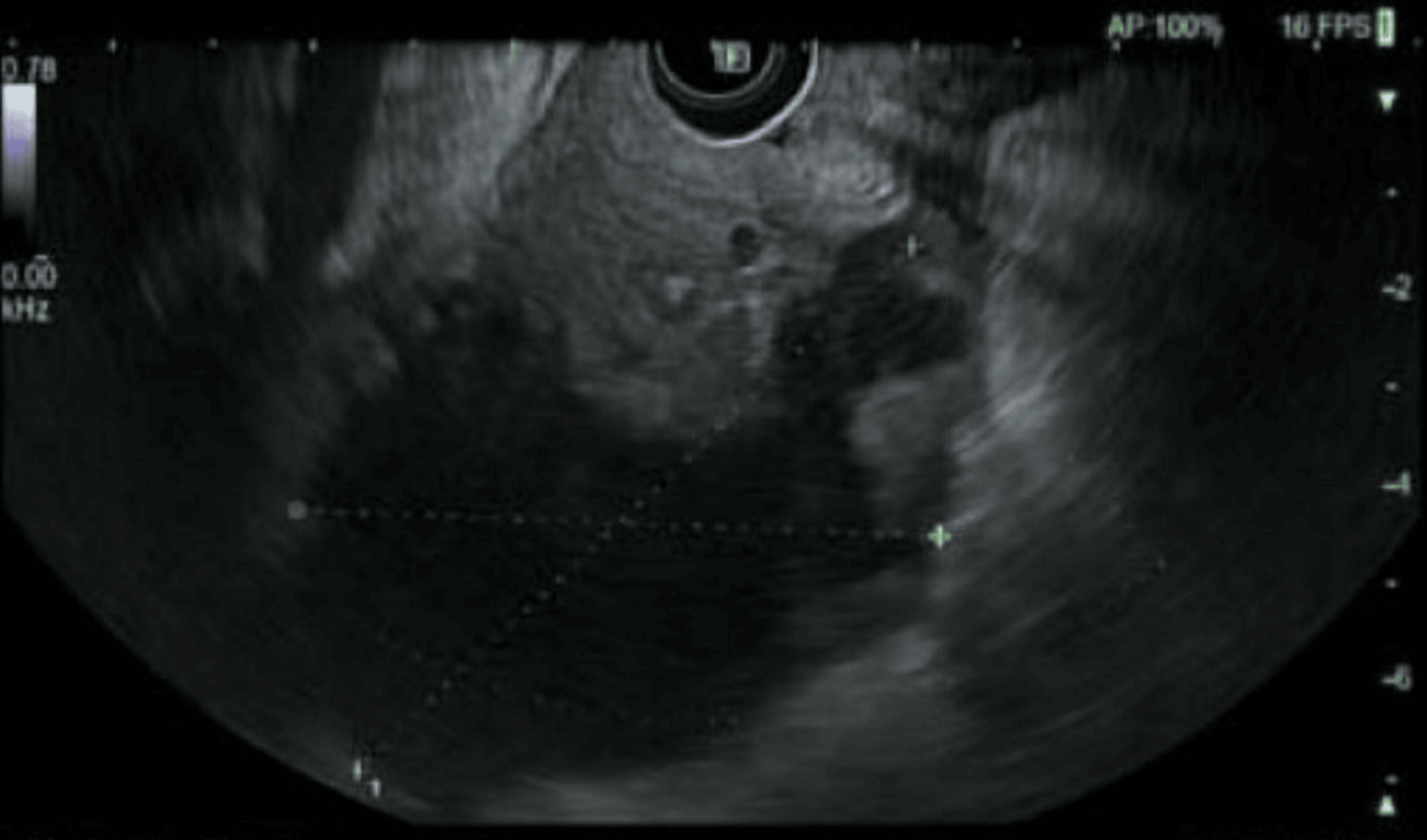
Endoscopic ultrasound of duodenal mass with hypoechogenicity and septations

EUS was then performed, demonstrating no abnormalities of the common bile duct, main pancreatic duct, or ampulla. However, imaging demonstrated a round intramural cystic lesion at the third portion of the duodenum that was hypoechoic with septations and measured approximately 6.0 x 7.6 cm in diameter (Figure 2). Diagnostic needle aspiration revealed a bloody aspirate. The decision was made to forgo any further aspiration attempts at this time. A 10Fr nasojejunal feeding tube was then placed under direct visualization with the endoscope.

**Figure 3:**
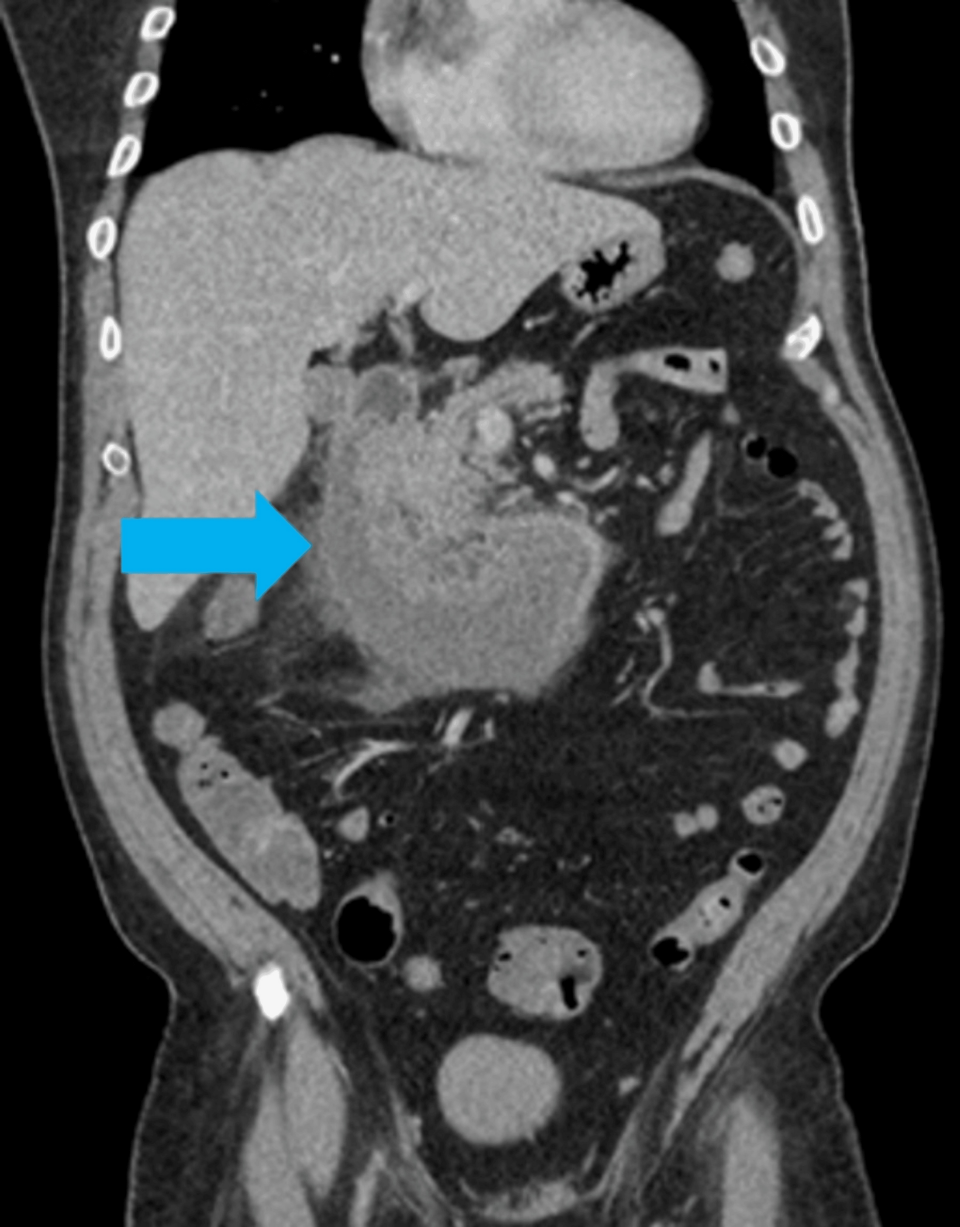
Computed tomography angiography (CTA) of the abdomen and pelvis, coronal view. Imaging performed one hour after completion of EGD and EUS re-demonstrated an intramural duodenal hematoma (blue arrow). EGD: esophagogastroduodenoscopy; EUS: endoscopic ultrasound

Post-procedural labs were obtained, which demonstrated a mild leukocytosis with hypokalemia and elevated lipase. CT angiography (CTA) of the abdomen and pelvis was also performed postoperatively to evaluate for any expansion of the hematoma after needle aspiration. The imaging study demonstrated a large C-shaped intramural/periduodenal fluid collection with rim enhancement, measuring 12.4 x 10.6 x 6.5 cm, extending from the proximal duodenum to the distal horizontal segment with possible breach of the inferior serosal wall (Figure 3). The patient remained nil per OS (NPO) and underwent resuscitation overnight with serial abdominal exams.

**Figure 4:**
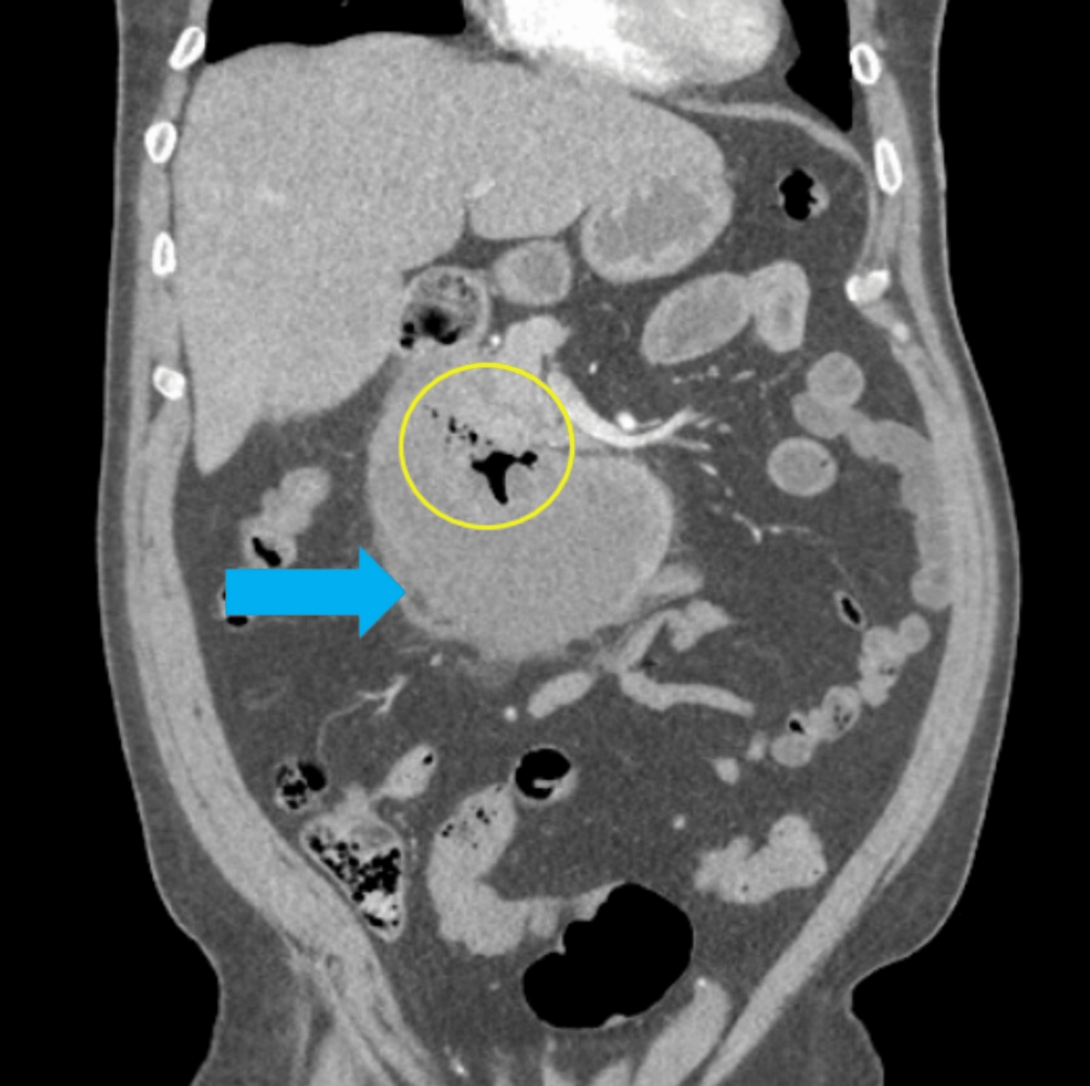
Computed tomography angiography (CTA) of the abdomen and pelvis, coronal view, lung window. Imaging performed 16 hours after endoscopic evaluation demonstrated an enlargement of the duodenal hematoma (blue arrow) in addition to a new cluster of gas bubbles (yellow circle).

On hospital day one, the patient’s abdomen continued to remain soft without evidence of peritonitis and was hemodynamically stable. Morning labs demonstrated a reactive leukocytosis of 20.9 x 10^3^/ul, hemoglobin of 14.5 g/dl, potassium of 3.2 mmol/l, with magnesium of 2.6mg/dl, and improvement in lipase. Unfortunately, the patient was trialed on oral intake and had immediate return of abdominal pain, and a repeat CTA abdomen and pelvis was performed. The new imaging redemonstrated that the intramural collection was slightly larger, 7.6 x 12 cm, with an associated cluster of gas bubbles noted centrally within the superior aspect of the collection, with no active bleeding, in addition to increased straining extending into the right hemipelvis (Figure 4). The patient continued to be hemodynamically stable; however, the decision was made to transfer the patient to a facility with interventional radiology capabilities for possible drainage. On hospital day two, the patient's leukocytosis and lipase were improving, and hemoglobin was stable. The nasojejunal feeding tube was placed, and the patient was ultimately transferred to a tertiary center in stable condition.

At the tertiary facility, the patient continued with nasojejunal decompression and was started on TPN. He underwent a repeat EGD and EUS within a week of transfer to the tertiary facility, with findings demonstrating stability of the intramural hematoma’s size; however, there was evidence of a duodenal stricture, and an AXIOS stent (Boston Scientific, Marlborough, Massachusetts, US) was placed in the second portion of the duodenum. After 21 days in the tertiary hospital, he was discharged home on a clear liquid diet with TPN supplementation and followed up in six weeks for stent removal. Unfortunately, he developed duodenal stenosis secondary to ulceration, resulting in a gastric outlet obstruction. He ultimately underwent a robotic gastrojejunostomy for duodenal bypass by a hepatobiliary surgeon approximately eight months after initial presentation to our community hospital.

## Discussion

Duodenal hematomas are a rare cause of upper gastrointestinal bleeding and obstruction. They can be caused by a multitude of factors, including blunt trauma, endoscopic procedures, bleeding disorders, and pancreatitis, with the most common cause being blunt abdominal trauma. The pathophysiology is due to the accumulation of blood within the wall of the duodenum, which can cause destruction of vessels within the submucosa; as such, the localized collection of blood can cause obstructive symptoms within the duodenum [3]. 

The initial presentation of a duodenal hematoma is often nonspecific, with symptoms ranging from vague abdominal pain to symptoms consistent with a small bowel obstruction [2]. Compared to traumatic intramural duodenal hematomas, which often develop at the subserosal layer of the duodenum, spontaneous intramural duodenal hematomas develop at the level of the duodenal mucosa or submucosa layers [1]. While the etiology is still unclear, there is a belief that the underlying duodenal vasculature is compromised from the release of pancreatic proteolytic enzymes, leading to hematoma formation [1,4]. Duodenal hematomas are typically diagnosed through the use of computed tomography (CT) scans, EGDs, and endoscopic ultrasounds [3].

It is well known that duodenal hematomas are often a result of trauma; however, spontaneous intramural duodenal hematomas are often associated with abnormalities in coagulation. If a patient has a spontaneous intramural duodenal hematoma with normal coagulation studies, pancreatic etiologies should be further investigated [5]. Common etiologies of the pancreas include hypercalcemia, biliary pathologies, alcohol use, steroids, hypertriglyceridemia, autoimmune diseases, and endoscopic interventions. Eurboonyanun et al. presented a case of a 27-year-old male with a spontaneous duodenal hematoma without evidence of trauma, with a heteroechoic mass at the posterolateral wall of the duodenum on endoscopic ultrasound [5]. The patient was found to have a significant alcohol history, 500 ml of liquor per day, and the development of his duodenal hematoma was a result of his alcoholic pancreatitis [5].

The mainstay of treatment for an intramural duodenal hematoma is non-operative management. This often includes nasogastric tube decompression with nutritional supplementation. Most initial treatment regimens include total parenteral nutrition with fluid replacement and NPO status [2,6]. Once there is a decrease in the size of the duodenal hematoma, a feeding tube can be passed distally to the hematoma to initiate enteral nutrition and eventually have the diet advanced as tolerated. Most patients have resolution of symptoms within one to three weeks. While there are no case reviews regarding a treatment algorithm for spontaneous duodenal hematomas, Kim et al. performed a literature review evaluating a nonoperative approach in the management of traumatic intramural duodenal hematomas [7]. Their review identified that the average resolution time was 7.69 days and that a nonoperative approach is not inferior to an operative intervention [6].

In the event that a duodenal hematoma does not resolve with conservative measures, more invasive procedures can be explored. Current indications for intervention include compression of the bile duct with the development of jaundice, intra-abdominal bleeding, or peritonitis [8,9]. The types of interventions are often dictated by patient stability and hospital capability. In regard to our patient, we lacked interventional radiology capability, and the patient was ultimately transferred out. At the tertiary facility, they had an AXIOS stent placed due to a duodenal stricture from the hematoma. This endoscopic intervention aligns with current literature for pursuing minimally invasive procedures first, which is also seen in the study by Lee et al., who performed endoscopic decompression for a duodenal hematoma caused by acute pancreatitis [10]. If unable to decompress endoscopically or if there are changes in patient presentation, surgical intervention such as hematoma evacuation or possible bypass can be considered. As seen in our patient, there was a complication from the stent resulting in ulceration and gastric outlet obstruction, requiring surgical bypass. While there are no cases in the literature that illustrate an intramural hematoma requiring both endoscopic and surgical intervention, Elmoghazy et al. discussed an exploratory laparotomy on a patient with an idiopathic duodenal hematoma who developed peritonitis and free air on chest x-ray. Intra-operatively, they encountered hemoperitoneum with an associated duodenal perforation and subsequently performed a duodenal exclusion gastrojejunostomy, gastroduodenal artery ligation, cholecystectomy, and a controlled duodenal fistula [9]. While there is no standard algorithm for the treatment of duodenal hematomas, patient symptomology is what dictates the types of intervention warranted.

## Conclusions

Duodenal hematomas are a unique manifestation, often as a result of trauma. However, more recent literature illustrates how the incidence of duodenal hematomas can be attributed to other factors such as anticoagulation or alcoholic pancreatitis. Despite the etiology, the treatment algorithm starts with conservative management to include NPO status and supplemental nutrition if warranted. Failure of conservative management dictates escalation of treatment beginning with endoscopic procedures; however, these interventions are not without risks, such as duodenal ulceration necessitating surgical bypass as seen in our patient. As such, each case of non-traumatic duodenal hematomas presents a unique opportunity to tailor treatment based on the patient's etiology, symptomology, and treatment facilities' capabilities.
